# Association of social isolation, loneliness and risk of cardiovascular diseases: Meta-analysis of cohort studies

**DOI:** 10.1186/s12889-025-24300-z

**Published:** 2025-09-24

**Authors:** Liaoyao Wang, Hejing Pan, Ziling Cai, Xuanlin Li, Le Luo

**Affiliations:** 1 Chinese Medicine Department, Hangzhou Wuyunshan Hospital (Hangzhou Institute for Health Promotion), Hangzhou, 310008 China; 2 School of Basic Medical Sciences, Zhejiang Chinese Medical University, Hangzhou, 310053 China

**Keywords:** Social isolation, Loneliness, Cardiovascular diseases, Meta-analysis, Cohort

## Abstract

**Background:**

The association between social isolation, loneliness and risk of cardiovascular diseases (CVD) is not fully understood. This meta-analysis aims to explore social isolation and loneliness whether increases the risk of CVD.

**Methods:**

Data sources was PubMed and Embase from inception to 10 February 2025. The risk of bias was assessed using the Newcastle-Ottawa Quality Assessment Scale (NOS). Hazard ratios (HR) with 95% confidence intervals (CI) were pooled using a random-effect model, and publication bias was assessed with funnel plots and Egger’s test.

**Results:**

This meta-analysis included six cohort studies with a total of 5,253,128 participants, spanning a follow-up period of 4 to 11.3 years from publications between 1996 and 2022. All studies were of high quality (NOS score ≥ 7). The pooled analysis revealed a heightened risk of CVD among individuals experiencing social isolation or loneliness (HR = 1.17, 95% CI 1.10–1.25, I^2^ = 85.1%, *P* < 0.001). Subgroup analysis indicated that patients with a history of social isolation had a slightly higher risk of CVD compared to those with loneliness [HR = 1.39, 95% CI 1.15–1.68, I^2^ = 90.2%, *P* = 0.001]. Additionally, the risk of CVD was slightly elevated during the 4–7 year follow-up compared to 7–9 years and 10–11 years [HR = 1.87, 95% CI 1.67–2.10, I^2^ = 0%, *P* < 0.001]. Those with a history of social isolation or loneliness had the highest risk of stroke [HR = 1.23, 95% CI 1.07–1.43, I^2^ = 74.5%, *P* = 0.004]. Furthermore, Asian populations exhibited a slightly higher risk of CVD compared to North American and European populations [HR = 1.46, 95% CI 1.12–1.91, I^2^ = 0%, *P* = 0.005].

**Conclusions:**

The increased risk of CVD among social isolation or loneliness individuals underscore the importance of prioritizing their care in clinical practice and nursing. However, the high heterogeneity in meta-analysis suggests the need for further studies to validate and explore this association thoroughly.

**Supplementary Information:**

The online version contains supplementary material available at 10.1186/s12889-025-24300-z.

## Background

Cardiovascular diseases (CVD) are among the most common chronic conditions worldwide, including coronary heart disease (CHD), stroke, heart failure (HF), and acute myocardial infarction (AMI). They have become a major global public health issue, with rising mortality rates and disease burden [1]. According to the World Health Organization, CVDs account for approximately 18 million deaths annually, remaining the leading cause of global mortality [2]. The 2022 Report on Cardiovascular Health and Disease in China indicated that modifiable risk factors such as smoking, unhealthy eating habits, physical inactivity, increased body mass index, and psychological factors impact cardiovascular health[3]. Therefore, in order to effectively reduce the burden and mortality of CVDs in individuals and populations, it is essential to explore the relationship between cardiovascular risk factors and disease progression. Despite significant advances in medical treatment and preventive strategies, psychosocial factors play a crucial role in cardiovascular health. Unhealthy lifestyles, such as smoking, alcohol consumption, and physical inactivity, may be associated with social isolation [4]. Research has shown that individuals with social isolation or a history of loneliness face higher mortality risks compared to those with strong social networks [5], This phenomenon has sparked increased attention to the impact of social isolation and loneliness on cardiovascular health.

Social isolation refers to a lack of social contact or integration within a social network, while loneliness is the subjective distress caused by perceived isolation [6]. As social interaction increasingly shifts to digital communication, with a decrease in face-to-face interactions, loneliness has become an escalating global issue [7]. Social isolation has been linked to adverse health outcomes such as depression, stress, and cognitive decline [8]. Chronic loneliness may lead to cardiovascular risk factors, including elevated blood pressure and inflammatory markers [9]. Although social isolation and loneliness are distinct, they share common pathways in their negative impact on health and may contribute to the development of cardiovascular diseases [10]. Existing studies have confirmed an association between social isolation or loneliness and cardiovascular diseases, but current evidence primarily focuses on mortality risk, with limited research on incidence risk [11, 12]. A meta-analysis has shown that social isolation and loneliness increase the risk of coronary heart disease and stroke [13], but the association with other types of cardiovascular diseases has not been reported. An 8-year cohort study found that elderly women with social isolation and loneliness had a higher risk of CVD. However, the limitations of the study sample also restricted the generalizability of the findings [14]. A 2022 systematic review based on populations in Australia and New Zealand reported no association between social isolation, loneliness, and the incidence of CVD [15]. Currently, there is no unified conclusion regarding the relationship between the two. This study will conduct a systematic review of existing population-based cohort studies to assess the impact of loneliness and social isolation on cardiovascular diseases. Through this meta-analysis, we aim to contribute to the evidence base regarding loneliness and social isolation as public health issues and advocate for including social isolation and loneliness management in cardiovascular disease prevention and intervention strategies.

## Methods

This meta-analysis registered with PROSPERO (CRD42024517774), adheres to the Preferred Reporting Items for Systematic Review and Meta-Analysis (PRISMA 2020) [16] statement.

### Data sources

PubMed and EMBASE were searched without any restrictions from inception to 10 February 2025. The subject terms (Emtree in Embase, MeSH in PubMed) and corresponding keywords were used. Search terms included those related to social isolation, loneliness, and cardiovascular diseases and its variants. The reference lists of retrieved studies and previous meta-analyses were also checked to identify other studies that might be eligible for inclusion. The full search strategy for these databases is provided in Supplementary Tables 1–2.

### Study selection

The retrieved initial records were imported into Endnote software, and duplicate records were removed. In total, two authors (Hejing Pan and Ziling Cai) independently reviewed titles and abstracts to exclude irrelevant records and then classified the remaining records according to inclusion, exclusion, or uncertainty. For records that were uncertain, the full text was read to ensure eligibility for inclusion. Any disputes were resolved through group discussion.

### Eligibility criteria

Studies were included if they meet following criteria: (a) Outcomes: patients with CVD; (b) Exposure: patients experiencing social isolation (Social isolation refers to the condition where an individual lacks social connections or interactions with others, often characterized by a lack of family, friends, or social support networks) or loneliness (Loneliness is an emotional experience of feeling a lack of or dissatisfaction with social relationships, even when surrounded by others); (c) Comparator: healthy people or non-social isolation/non-loneliness sufferers; (d) Effect size: hazard ratios (HRs) and relative risks (RRs); and (e) Study type: cohort study.

The exclusion criteria were as follows: (a) conference abstracts, letters to editors or reviews; (b) duplicate publication; and (c) incomplete data or no interested outcome.

### Data extraction

For each eligible study, two reviewers (Liaoyao Wang and Hejing Pan) independently extracted relevant data using Microsoft Excel software. Disagreements were resolved through discussion or adjudication by a third reviewer (Xuanlin Li). Data extracted included study characteristics (author, publication year, country), participant demographics (sample size, outcomes, age), follow-up duration, diagnostic criteria, adjusted confounders, and effect sizes of associations between social isolation, loneliness, and cardiovascular event risk.

### Study quality

The Newcastle-Ottawa Quality Assessment Scale (NOS) [17] was employed to assess the quality of the included studies across three domains: selection, comparability, and outcomes. It incorporates criteria pertaining to participant selection (4 points), comparability between groups (2 points), and exposure factor measurement (3 points). Cohort study scores ranged from 0 to 9 stars, with higher scores indicating superior quality. Studies with NOS stars ≥ 7, 4–6, and 0–3 were classified as high, moderate, and low quality, respectively.

### Data analysis

The Stata software (version 18) was used to conduct the data analysis. We assessed heterogeneity using the chi-square test and I^2^ value, and *P* < 0.1 or I^2^ > 50% indicated that heterogeneity was very high, and thus, the random-effects model was adopted. We performed sensitivity analysis to test the robustness of the meta-analysis results and used the one-by-one elimination method to explore the sources of heterogeneity. Subgroup analysis was conducted according to the type of exposure, follow-up time, area and the type of CVD. The publication bias was tested by using funnel plots, egger’s regression tests.

## Results

### Study selection

**Fig. 1:**
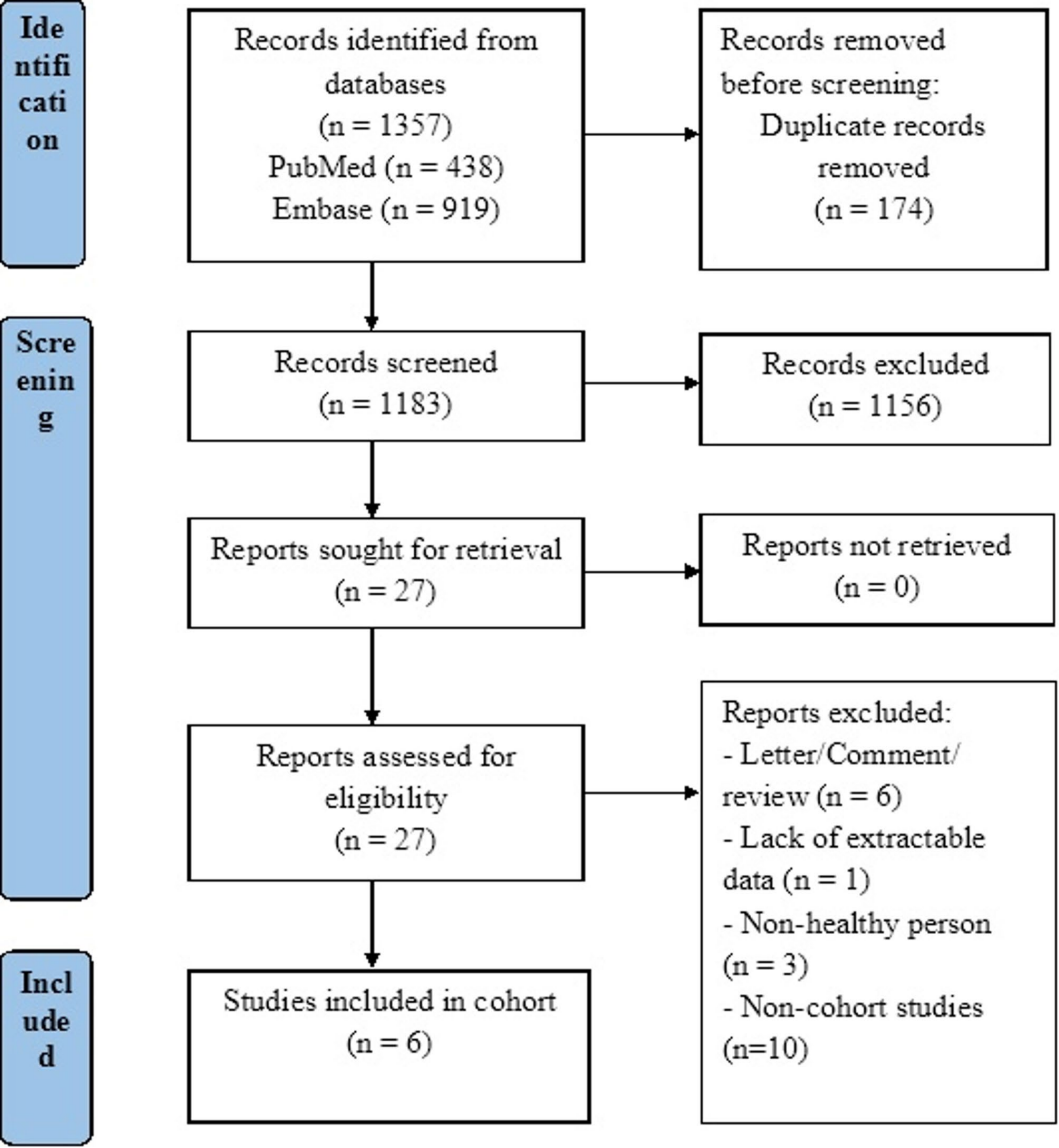
PRISMA flow diagram of studies included in the cohort

Initially, 174 duplicate citations were removed. Following title and abstract screening, 27 full-text articles were evaluated, resulting in 6 eligible studies. The flow diagram in Fig. 1 outlines the literature screening process.

### Study characteristics

Table 1 summarizes the characteristics of the 6 included cohort studies [11, 14, 18–21], all published between 1996 and 2022. The sample sizes ranged from 32,624 to 3,700,915, with follow-up periods ranging from 4 to 11.3 years. CVD were primarily diagnosed according to the “International Classification of Diseases (ICD)” standard, while social isolation and loneliness were typically assessed using the Berkman-Syme social networks index and the 3-item University of California, Los Angeles (UCLA) Loneliness Scale, respectively. Three studies [11, 18, 20] examined the association between social isolation and cardiovascular event risk, one study [19] focused on loneliness, and two studies [14, 21] explored both social isolation and loneliness in relation to CVD. Adjusted confounders varied slightly across studies, with age, sex, ethnicity, body mass index (BMI), smoking, alcohol consumption, and physical activity being the most commonly adjusted confounders.

**Table 1:** Characteristics of studies included in the meta-analysis

References	Country	Sample size	No. of outcomes	Follow-up years	Diagnostic of exposure	Diagnostic of outcomes	Age	Confounders adjusted
Kawachi, et al. (1996) [20]	American	Total: 32,624 Social isolation: 15,823 Non-social isolation: 16,801	1087	4 years	Berkman-Syme social networks index	ICD-9 codes	42–77	Age, cigarette smoking, alcohol intake, body mass index, history of hypertension, diabetes mellitus, hyper cholesterolaemia, angina pectoris, family history of myocardial infarction before age 60, and physical activity.
Li, et al. (2022) [19]	China	Total: 3,700,915 Loneliness: 620,816 non-loneliness: 3,080,099	4283	11.3 years	Self-reported	ICD-10 codes	40–69	Age, sex, ethnic, Townsend Deprivation index, educational level, body mass index, average total annual household income, physical activity, smoking, alcohol consumption, healthy diet score, total cholesterol, high-density li proprotein cholesterol, hypertension, diabetes, family history of heart diseases or stroke.
Golaszewski, et al. (2022) [14]	Canada	Total: 57,825 Social isolation: 25,130 Non-social isolation: 32,695 Loneliness: 20,062 non-loneliness: 37,763	1599	9 years	The 3-item UCLA Loneliness Scale	ICD-10 codes	65–99	Age, race and ethnicity, educational level, history of depression, health behaviors (smoking, alcohol consumption, and physical activity); and variables (history of diabetes, hypertension medication use, hyperlipidemia medication use, general health, and physical functioning).
Hakulinen, et al. (2018) [21]	Finland	Total:479,054 Social isolation: 42,595 Non-social isolation: 427,709 Loneliness: 28,513 non-loneliness: 428,722	AMI:5731 Stroke:3471	7.1 years	Scales that were used in a previous UK Biobank study/Revised UCLA Loneliness Scale	ICD-10 codes	40–69	Age, sex and ethnicity
Smith, et al. (2021) [11]	UK	Total:938,558 Social isolation: 559,527 Non-social isolation:379,031	CHD:42,402 Stroke:19,999	7 years	Berkman-Syme social network index	ICD-10 codes	63–68	Age, sex, region of recruitment, deprivation, smoking, alcohol intake, physical activity, body-mass index, and self-rated health
Ikeda, et al. (2008) [18]	Japan	Total:44,152 Social isolation: 4249 Non-social isolation: 39,903	CHD:301 Stroke:1057	10.7 years	Existing social support scales	ICD-10 codes	40–69	Age, smoking status, ethanol intake, body mass index, leisure time sports activity, perceived stress and occupation.

Abbreviations: *UK* United Kingdom, *CVD* Cardiovascular disease, *CHD* Coronary heart disease, *AMI* Acute myocardial infarction

### Quality assessment

All six cohort studies assessed quality using the NOS scale, with scores ≥ 7. Specifically, two studies [18, 19] scored 9 points, two studies [11, 14] scored 8 points, and two studies [20, 21] scored 7 points, indicating high-quality studies for inclusion in this meta-analysis. Detail of quality assessment is provided in Table 2.

**Table 2:** Quality of cohort studies

Study	Year	Selection	Comparability	Outcome	Overall quality score
Cohort studies (*n* = 6)					
Kawachi, et al.	1996	***	**	**	7
Li, et al.	2022	****	**	***	9
Golaszewski, et al.	2022	****	**	**	8
Hakulinen, et al.	2018	***	*	***	7
Smith, et al.	2021	***	**	***	8
Ikeda, et al.	2008	****	**	***	9

### Social isolation, loneliness and risk of CVD

**Fig. 2:**
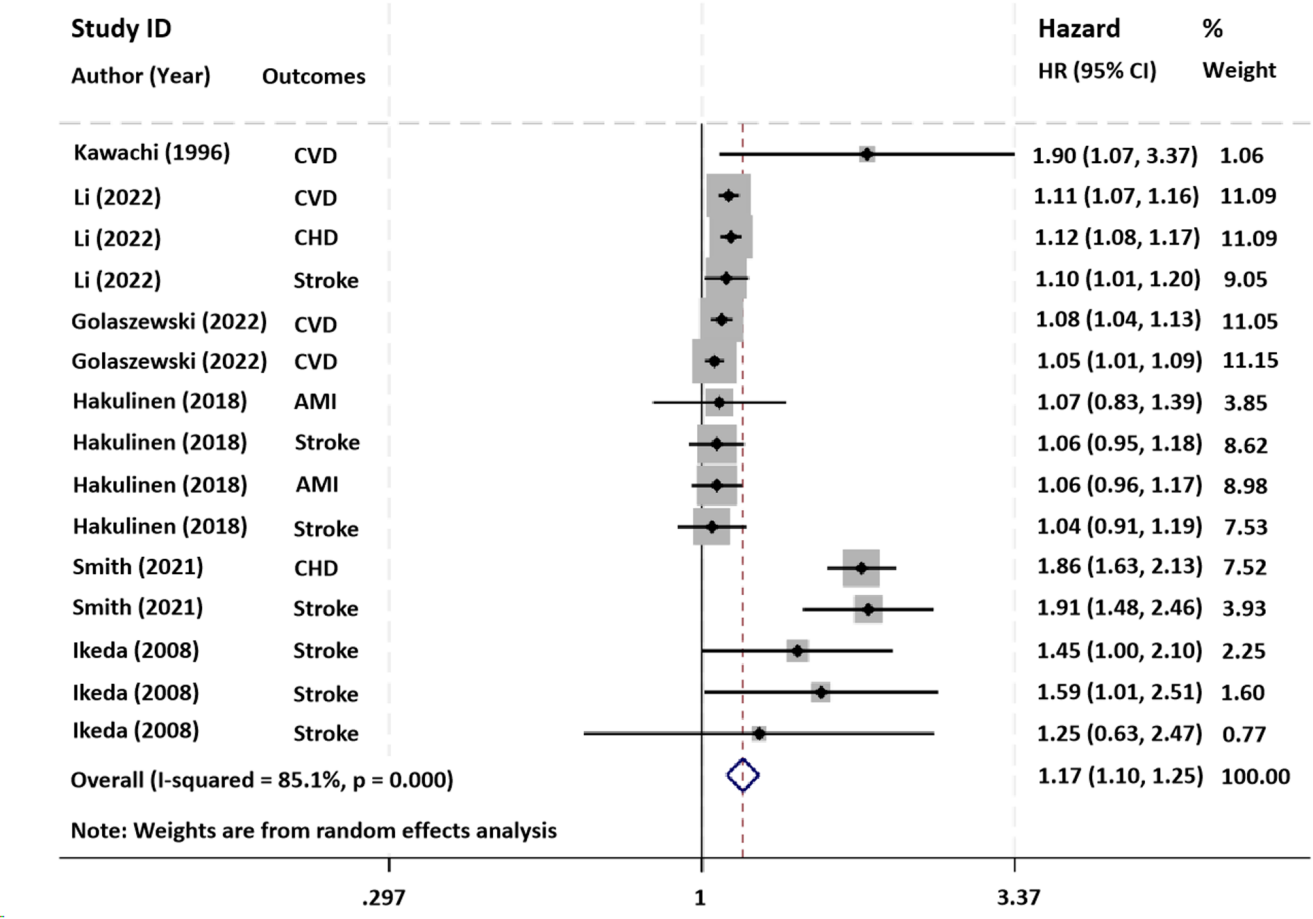
Forest plot for the risk of CVD in social isolation and loneliness groups. Abbreviations: HR, Hazard ratio; CI, Confidence interval; CVD, Cardiovascular disease; CHD, Coronary heart disease; AMI, Acute myocardial infarction

Six cohort studies [11, 14, 18–21] explored the association between a history of social isolation or loneliness and the risk of CVD. The pooling analysis shows that a history of social isolation or loneliness is associated with an increased risk of CVD [HR = 1.17, 95%CI 1.10–1.25, I^2^ = 85.1%, *P* < 0.001, Fig. 2]. Sensitivity analysis showed that none of the individual studies reversed the pooled-effect size, which means that the results are robust (Supplementary Fig. 1).

### Subgroup analysis

Subgroup analyzes were conducted based on the type of exposure, follow-up time, the type of CVD and area. In the subgroup analysis, patients with a history of social isolation had a slightly higher risk of CVD than those with a history of loneliness [HR = 1.39, 95%CI 1.15–1.68, I^2^ = 90.2%, *P* = 0.001], and the risk of CVD was slightly higher in follow-up years 4–7 than in years 7–9 and 10–11 [HR = 1.87, 95%CI 1.67–2.10, I^2^ = 0%, *P* < 0.001], patients with a history of social isolation/loneliness had the highest risk of Stroke [HR = 1.23, 95%CI 1.07–1.43, I^2^ = 74.5%, *P* = 0.004], and Asian populations had a slightly higher risk of CVD than North American States and Europe [HR = 1.46, 95%CI 1.12–1.91, I^2^ = 0%, *P* = 0.005] (Table 3).

**Table 3:** Results of subgroup analysis

Measures	Pooled hazard ratio (95% CI)	*P*
Exposure		
Social isolation	1.39 (1.15,1.68)	0.001
Loneliness	1.09 (1.06,1.12)	< 0.001
Follow-up years		
4–7, y	1.87 (1.67,2.10)	< 0.001
7–9, y	1.06 (1.04,1.09)	< 0.001
10–11, y	1.12 (1.09,1.15)	< 0.001
CVD		
CVD	1.08 (1.04,1.13)	< 0.001
AMI	1.06 (0.97,1.16)	0.207
Stroke	1.23 (1.07,1.43)	0.004
CHD	1.44 (0.87,1.43)	0.153
Area		
North America	1.07 (1.01,1.13)	0.016
Europe	1.19 (1.09,1.30)	< 0.001
Asia	1.46 (1.12,1.91)	0.005

Abbreviations: *HR* Hazard ratio, *CI* Confidence interval, *CVD* Cardiovascular disease, *CHD* Coronary heart disease, *AMI* Acute myocardial infarction

### Publication bias

**Fig. 3:**
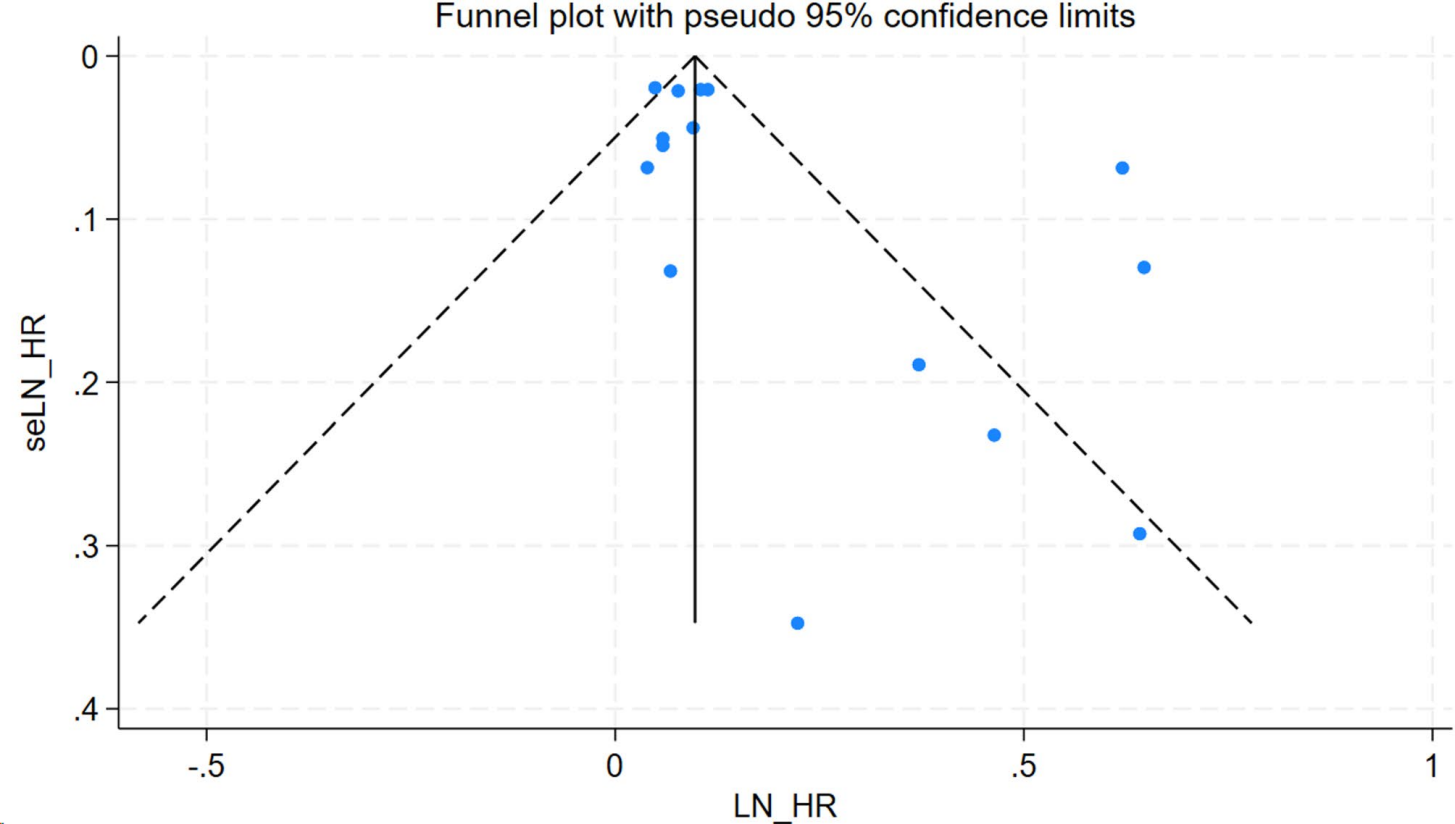
Funnel plot for risk of CVD in social isolation and loneliness groups

A visual inspection of the funnel plot showed no evidence of a significant publication bias in the outcome of social isolation/loneliness and risk of CVD (Fig. 3). Egger’s regression test (*P* = 0.058 > 0.05) likewise indicated no publication bias in our meta-analysis.

## Discussion

### Principal findings

The meta-analysis included 6 cohort studies with a total of 5,253,128 individuals. The result indicated that the risk of CVD in patients experiencing social isolation or loneliness was 1.17-fold higher than in patients not experiencing social isolation or loneliness.

### Comparison with previous studies

Freak et al. [15] conducted a systematic review and meta-analysis examining the relationship between social isolation, lack of social support, or loneliness and the risk of CVD. Although their findings indicated no association between social health and CVD incidence, our study revealed a significant association. This discrepancy may stem from multiple factors. First, Freak et al.‘s study relied on self-reported CVD diagnoses, which could introduce diagnostic bias. In contrast, our study employed a more standardized approach by using ICD-10 codes to identify CVD, thereby reducing diagnostic bias and the impact of confounding factors. Additionally, Freak et al. did not conduct a detailed analysis of follow-up duration, whereas our study shows that follow-up time significantly influences the heterogeneity of the results. In our meta-analysis, longer follow-up durations revealed a stronger association between social isolation and loneliness with CVD risk, suggesting that differences in follow-up years may be an important factor contributing to the inconsistency in results. Second, Freak et al.‘s study included a limited number of cohort studies and lacked comprehensive CVD outcome data, which restricted the generalizability of their conclusions. Our study focused on a broader set of cohort studies and included various CVD outcomes such as heart disease and stroke, enabling a more comprehensive evaluation of the relationship between social isolation and CVD. Furthermore, the geographic scope of Freak et al.‘s study was relatively narrow, primarily focusing on populations in Australia and New Zealand, which may not capture the diverse cultural and lifestyle factors affecting cardiovascular health in other regions. In contrast, our study encompassed populations from North America, Europe, and Asia, which not only enhanced the generalizability of the results but also allowed for subgroup analyses that further revealed the impact of regional cultural differences on CVD risk. For instance, Asian populations exhibited a higher risk in the relationship between social isolation and CVD, likely influenced by regional culture, social support systems, and lifestyle factors.

Additionally, Albasheer et al. [22] published research focusing on the impact of social isolation and loneliness on cardiovascular disease risk factors. Although our study overlaps with some of their findings, we included a broader range of observational studies, covering cohort studies with various designs. This helped reduce heterogeneity and methodological bias, making our results more robust. In contrast, although Albasheer et al.‘s study included data from multiple countries, the geographic distribution was not clearly specified, which may limit the generalizability of their findings. Our study performed subgroup analyses with explicit geographic region classifications, further revealing the higher risk among Asian populations and highlighting the important role of regional culture in cardiovascular disease. Finally, our study integrated mechanistic research and proposed targeted intervention strategies, providing more practical guidance for clinicians. By exploring how social isolation and loneliness increase CVD risk through physiological mechanisms such as stress and inflammation, we are able to offer more tailored intervention suggestions for different populations, an aspect that was not sufficiently explored in previous studies. In conclusion, while the studies by Freak et al. [15] and Albasheer et al. [22] offer valuable perspectives, our study presents different conclusions due to differences in sample sources, methodology, follow-up duration, and cultural background. Through more comprehensive cohort studies, longer follow-up periods, broader geographic distribution, and in-depth mechanistic research, our study provides more robust evidence for the relationship between social isolation, loneliness, and CVD risk, as well as useful guidance for the development of clinical intervention strategies.

### Interpretation of findings

To date, extensive research has been conducted on the role of social isolation or loneliness in the risk of other diseases [23–25]. However, the role of social isolation or loneliness in CVD risk remains debated. Prior research [26–28] has primarily addressed their impact on CVD mortality and overall mortality. For instance, a meta-analysis of 90 cohort studies revealed that social isolation and loneliness elevate the risk of death and all-cause mortality among cardiovascular patients [29]. However, our meta-analysis shifts the focus to the association between social isolation or loneliness and incident cardiovascular disease. Our findings indicate a heightened risk of CVD among individuals experiencing social isolation or loneliness compared to their counterparts. Furthermore, subgroup analyses, including exposure factors, follow-up duration, CVD, and geographic regions, revealed several key insights: (1) Social isolation poses a greater risk for CVD compared to loneliness; (2) Longer follow-up durations contribute to increased heterogeneity in results; (3) While social isolation or loneliness is linked to heightened risks of CVD and stroke, it shows no association with CHD and AMI, consistent with previous research; (4) The risk elevation associated with social isolation or loneliness is more pronounced among Asians compared to North American and European populations, possibly reflecting differences in social well-being. These findings offer valuable implications for clinicians and CVD patients: prioritizing routine screening for social isolation or loneliness and interventions to enhance social connectedness is crucial. Moreover, recognizing the role of poor social health in CVD underscores the need for guidelines addressing the health risks of social isolation and loneliness, thereby aiding in CVD prevention and treatment efforts.

Social isolation is often associated with unhealthy behavioral changes, such as physical inactivity, irregular diet, and insufficient sleep, all of which have been shown to be significantly linked to CVD. Additionally, individuals experiencing social isolation may lack adequate social support and access to healthcare resources, leading to delayed intervention for health issues. In contrast, loneliness is more closely related to psychological stress, which affects cardiovascular health through mechanisms such as the activation of the body’s stress response, increased inflammation, and elevated cardiovascular burden. Evidence suggests that oxidative stress may be a key molecular mechanism linking chronic psychosocial stress to CVD. Therefore, reducing oxidative stress could serve as a therapeutic strategy to mitigate the harmful effects of social stress on health [30]. From a systems biology perspective, social isolation may amplify CVD risk through interactions among multiple organs. Research indicates that dysbiosis of the gut microbiome, leading to endotoxin (LPS) translocation, can exacerbate systemic inflammation, while long-term isolation-induced muscle atrophy may disrupt myocardial energy metabolism through abnormal secretion of myokines [31]. In contrast, the effects of loneliness are primarily focused on the “brain-heart axis,” with abnormal functional connectivity between the prefrontal cortex and limbic system potentially damaging the cardiovascular system indirectly by increasing psychological stress load [32]. Thus, it is evident that social isolation poses a greater risk than loneliness.

### Strengths and limitations

Our meta-analysis presents several strengths. It explores, for the first time, the association between social isolation or loneliness and CVD risk, utilizing a baseline of patients free from cardiovascular or other diseases to minimize confounding factors. Additionally, it was registered in PROSPERO and adhered to the updated PRISMA checklist, enhancing process transparency and result reliability. Additionally, the data we included were derived from cohort studies, which helps avoid the recall bias commonly found in retrospective studies, thanks to their clear documentation of the ‘exposure-outcome’ timeline. This assists in eliminating the interference of reverse causality and enhances our understanding of the relationship between cardiovascular diseases, loneliness, and social isolation. Cohort studies at baseline allow for the systematic evaluation of confounding factors and adjustments through multivariable models, which strengthens the robustness of the results, thereby providing more solid evidence for the prevention of cardiovascular diseases. However, potential limitations exist. The lack of standardized diagnostic criteria for social isolation and loneliness may have influenced the meta-analysis results. Due to the wide variety of inconsistent exposure measurement tools and the difficulty in categorizing them, subgroup analysis could not be performed, which limited the exploration of the sources of heterogeneity. Furthermore, statistical heterogeneity was observed, although sensitivity analyses yielded robust findings, impacting result reliability. Despite conducting a thorough literature search and excluding conference abstracts to mitigate biased methodological assessments, the possibility of missed studies remains a challenge.

## Conclusions

Our meta-analysis revealed an elevated risk of CVD in individuals experiencing social isolation and loneliness. This highlights the importance of prioritizing their care in clinical practice and care. However, the high degree of heterogeneity in the meta-analysis suggests that further research is necessary to validate and explore this association in depth.

## Supplementary Information

Below is the link to the electronic supplementary material.

Supplementary Material 1

## Data Availability

The data used and/or analyzed in the current study is available from the first author on reasonable request.

## Abbreviations

AMI: Acute Myocardial Infarction
CI: Confidence Interval
CHD: Coronary Heart Disease
CVD: Cardiovascular Diseases
HF: Heart Failure
HR: Hazard Ratios
ICD: International Classification of Diseases
NOS: Newcastle-Ottawa Quality Assessment Scale
UCLA: University of California, Los Angeles
PRISMA: Preferred Reporting Items for Systematic Review and Meta-Analysis
