# Supplementary material for: Association of social isolation, loneliness and risk of cardiovascular diseases: Meta-analysis of cohort studies
Source: BMC Public Health. 2025 Sep 24;25:3082. doi: 10.1186/s12889-025-24300-z (PMC12462301; doi:10.1186/s12889-025-24300-z)
Supplement: Supplementary file 1 — Supplementary Material 1 [file 12889_2025_24300_MOESM1_ESM.doc]

**Supplementary Material**

**Table S1~S2: Details of the Literature Search Strategy**

(1) PubMed ((Feb 10, 2025)

| **Search** | **Query** | **Items found** |
| --- | --- | --- |
| #1 | (Social isolation[Mesh]) OR (social segregation[Mesh]) OR (Loneliness[Mesh]) | 38,633 |
| #2 | (Social isolation[Title/Abstract]) OR (social loneliness[Title/Abstract]) OR (social segregation[Title/Abstract]) OR (Feeling isolated[Title/Abstract]) OR (social disaffiliation[Title/Abstract]) OR (Lonely[Title/Abstract]) OR (Loneliness[Title/Abstract]) | 29,620 |
| #3 | #1OR#2 | 57,218 |
| #4 | ("Coronary Disease"[Mesh]) OR ("Myocardial Ischemia"[Mesh]) OR ("Acute Coronary Syndrome"[Mesh]) OR ("Myocardial Infarction"[Mesh]) | 487,205 |
| #5 | (Coronary Disease[Title/Abstract]) OR (Coronary Heart Disease[Title/Abstract]) OR (Myocardial Ischemia[Title/Abstract]) OR (Ischemic Heart Disease[Title/Abstract]) OR (Acute Coronary Syndrome[Title/Abstract]) OR (Cardiovascular Stroke[Title/Abstract]) OR (Myocardial Infarct*[Title/Abstract]) OR (Heart Attack[Title/Abstract]) OR (Angina Pectoris[Title/Abstract]) OR (Stenocardia[Title/Abstract]) OR (Angor Pectoris[Title/Abstract]) OR (CHD[Title/Abstract])) OR (CAD[Title/Abstract]) | 450,376 |
| #6 | #4 OR #5 | 658,229 |
| #7 | #3 AND #6 | 447 |

(2) Embase (Feb 10, 2025)

| **Search** | **Query** | **Items found** |
| --- | --- | --- |
| #1 | 'Social isolation'/exp OR 'social loneliness'/exp OR 'social segregation'/exp OR 'Lonely'/exp OR 'Loneliness'/exp | 60,566 |
| #2 | 'Social isolation':ab,ti OR 'social loneliness':ab,ti OR 'social segregation':ab,ti OR 'Feeling isolated':ab,ti OR 'social disaffiliation':ab,ti OR 'Lonely':ab,ti OR 'Loneliness':ab,ti | 35,484 |
| #3 | #1 OR #2 | 61,355 |
| #4 | 'Coronary Disease'/exp OR 'Myocardial Ischemia'/exp OR 'Acute Coronary Syndrome'/exp OR 'Myocardial Infarction'/exp | 886,845 |
| #5 | 'coronary disease':ab,ti OR 'coronary heart disease':ab,ti OR 'myocardial ischemia':ab,ti OR 'ischemic heart disease':ab,ti OR 'acute coronary syndrome':ab,ti OR 'cardiovascular stroke':ab,ti OR 'myocardial infarct*':ab,ti OR 'heart attack':ab,ti OR 'angina pectoris':ab,ti OR stenocardia:ab,ti OR 'angor pectoris':ab,ti OR chd:ab,ti | 584,333 |
| #6 | #4 OR #5 | 1,045,575 |
| #7 | #3 AND #6 | 962 |


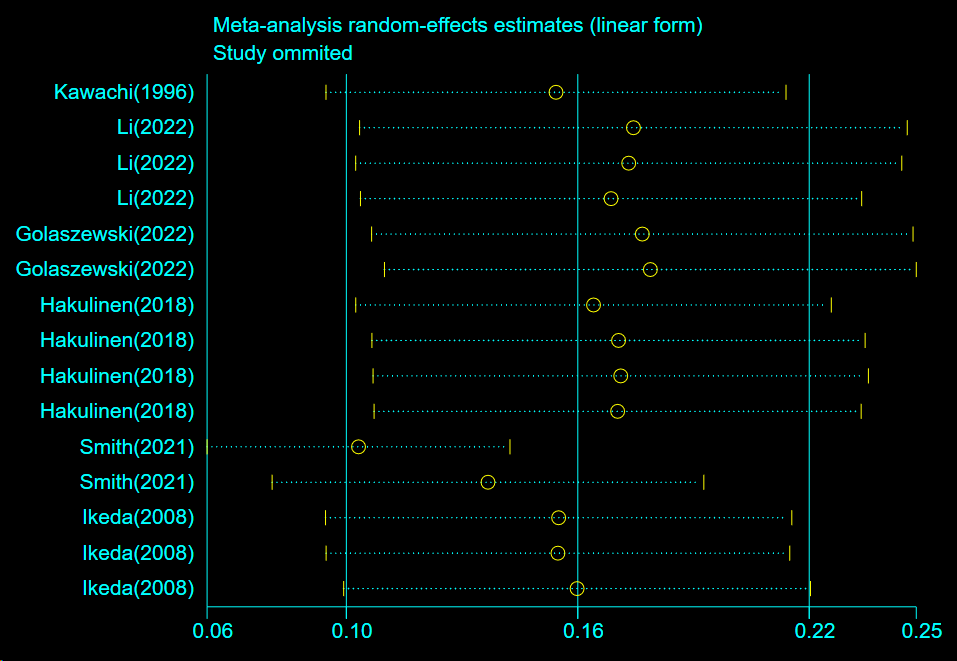


**Figure S1.** Sensitivity analysis for the risk of cardiovascular events in social isolation and loneliness.
